# A 5-year retrospective outlook of cardiovascular risk(s), outcome and survival peculiarities among patients in medical confinement; a tropical perspective

**DOI:** 10.4314/gmj.v58i4.2

**Published:** 2024-12

**Authors:** Titilope A Bamikefa, Peter K Uduagbamen, Olanrewaju O Olayemi, Olufemi O Ojewuyi, Oyelola Adeoye, Sekinat Bola-Oyebamiji, Olubukola Ala, Abosede G Adeyeye

**Affiliations:** 1 Renal Unit UniOsun Teaching Hospital Osogbo, Osun State/Department of Medicine College of Health Sciences Osun State University Osogbo; 2 Division of Nephrology and Hypertension, Department of Internal Medicine, Bowen University/Bowen University Teaching Hospital, Ogbomosho, Nigeria; 3 Department of Internal Medicine, UniOsun Teaching Hospital, Osogbo, Osun State/Department of Medicine College of Health Sciences Osun State University Osogbo; 4 Department of Surgery UniOsun Teaching Hospital, Osogbo, Osun State/Department of Surgery College of Health Sciences Osun State University Osogbo; 5 Department of Public Health, Bowen University Iwo, Osun State./Bowen University Teaching Hospital, Ogbomosho, Nigeria; 6 Department of Obstetrics & Gynecology UniOsun Teaching Hospital, Osogbo, Osun State/Department of Obstetrics & Gynecology College of Health Sciences Osun State University Osogbo; 7 Endocrine and Metabolism Unit UniOsun Teaching Hospital Osogbo, Osun State/Department of Medicine College of Health Sciences Osun State University Osogbo

**Keywords:** Cardiovascular risks, medical confinements, Outcome modifiers, Survival peculiarities

## Abstract

**Objective:**

The study was designed to evaluate the distribution of cardiovascular risk(s), outcome modifiers and survival peculiarities among medically confined patients.

**Design:**

Evaluated admission and discharge summaries of medically confined patients retroactively over 5 years.

**Setting:**

Medical wards, UniOsun Teaching Hospital Osogbo, Osun State, Nigeria.

**Participants:**

Two thousand three hundred and forty (2340) male and female patients aged between 16 and 108 years.

**Main outcome measures:**

Admission pattern, cardiovascular risk distribution, outcome and survival peculiarities.

**Results:**

The mean age of the respondents was 53.2 (18.3) years with male preponderance (52.0%). Non-infectious diseases predominated as the principal causes of medical confinements (82.9%). Cerebrovascular accident (13.5%), acute decompensation of chronic kidney disease (11.6%) and type 2 diabetes mellitus (6.0%) were the prominent causes of morbidity. The median duration of confinement was 6.0 days. The overall crude mortality rate was 14.3%, with the highest case fatality rate (27.2%) among those with neurological morbidities. Clinical outcome was statistically influenced by age (p= 0.004), occupation (p=0.02), duration of confinement (p=0.002) and morbidity stratification into infectious/non-infectious aetiologies (p=0.040) on regression analysis. The number of medical sub-specialties involved (p < 0.001), specialty affected (p<0.001), and yearly pattern of hospitalisation (p< 0.001) had a statistical influence on Kaplan Meier's survival plots.

**Conclusion:**

Hospital confinements underlined by infection/non-infection-related medical causes exhibit variable outcomes. The loop-sided frequencies of its causes remain worrisome because of the unending challenges plaguing effective healthcare delivery in the tropics.

**Funding:**

None declared

## Introduction

The development, implementation, and evaluation of effective population-centred health intervention(s) in any climate should ideally depend on outcomes derived from periodic surveys targeted at demystifying and understanding the burden and trend of prevalent diseases and the outcomes in such geographical space.^1^ Surveys relating to the trend and distribution of diseases with either infectious or non-infectious origin continue to be an ongoing engagement in both emerging and developed economies, with often divergent outcomes taking cognisance of geographical and population-defining peculiarities.^1^,^2^ Despite the perennial dynamics of medically defined illnesses globally with distinguishing geographical marks, locally piloted efforts are imperative to have a full grasp of the burden of infectious and non-infectious ailments of medical origin to give a vivid picture of the challenges encountered to strengthen health policy conceptualisation and actualisation further.^1^,^3^,^4^

Medical illnesses herald an impactful proportion of inhospital confinements worldwide, with variable disease outcomes based on geographical location, incipient socioeconomic characteristics, genetics, access to quality health care, and effective health care coverage. 3,4 Noninfection related medical ailments often running a nonremitting clinical course encompasses a wide array of clinical conditions ranging from diseases originating from cardiovascular dysfunction, diabetes mellitus, neoplasms as well as chronic obstructive airway diseases.^4^,^5^ A significant percentage, if not all, of these non-infection-related ailments share diverse but interrelated predispositions emanating from the inculcation of unwholesome practices as well as an ever-changing civilisation, which has given them more prominence globally.^2^,^4^,^5^

Emerging economies, including countries of tropical extraction, which had been ravaged historically by infection-related diseases and their clinical sequelae, now have an enormous burden of non-communicable medical conditions almost at the same level as the emergent economies that hitherto were the repository of non-infection-related medical ailments. 1,2,4,5 This worrisome trend has led to an astronomical increase in mortalities attributable to diseases of non-infectious aetiologies in the third world, including Nigeria, with catastrophic consequences owing to poorly developed or non-existent health care practises and or regulations.^5^,^6^ In Sudan, the recurring causes of mortality were malignancy, neurological diseases, and severe asthma, which were in higher frequencies compared to infectious diseases attributed to mortalities with gender imbalance.^7^ This contrasted with findings from Uganda, where mortality linked to infectious diseases predominated.^8^

In the Western world with high earning capacity, the proportion of in-hospital confinements attributable to medical aetiologies is approximately half of the values reported for countries with low earning capacities, most of which are in Sub-Saharan Africa.^4^,^6^ Divergent patterns and sequelae of medical ailments underlying in-hospital confinements have been elucidated in various parts of the African continent, with a staggering proportion attributable to non-infectious cardiovascular causes, further giving credence to the forecast of the continent's potential to tilt the epidemiological pendulum of cardiovascular disorders in some years to come.^6^,^7^,^8^,^9^,^10^ A prior review of medical confinements in this facility over a decade ago identified cerebrovascular accidents as the highest contributor to hospitalisation.^9^ This study was set up to determine if there has been a shift from the previously highlighted pattern of medical confinements and attempt to evaluate factors responsible for the observed deviations.

Globally, medical-related illnesses are the most endemic causes of mortality, with an unfavourable disposition in their distribution among African countries, which bear close to two-thirds of the global morbidity burden.^11^ With the huge contribution of the continent to the global medical mortality figure, understanding the outcome modifiers as well as survival peculiarities in medically confined adult patients, which this study was set up to address, will assist in the initiation of targeted plans designed to herald a decline in mortalities now and in the foreseeable future. A major proportion of studies on medical confinements in Nigeria focused on the causes of morbidity alone, with only a few evaluating the survival determinants, hence the need for this study to unravel the effect(s) of demographic and/or specific clinical parameters on the outcome and survival of these confined adult respondents.^9^,^10^,^12^

## Methods

The demographic characteristics and clinical attributes of patients hospitalised within the confines of the UniOsun Teaching Hospital medical wards over 5 years from January 2017 to December 2021 were retrospectively highlighted after a thorough evaluation of their admission, clinical and discharge summaries. UniOsun Teaching Hospital, Osogbo, a 200-bed tertiary health facility, is strategically surrounded by both secondary and tertiary health facilities near each other to meet the health needs of its populace, totalling approximately 11 million as well as its immediate and far neighbours. All patients admitted into the medical wards after thorough evaluation at the different portals of entry into the hospital (medicaloutpatient clinics and accident & emergency) and had their clinical diagnosis corroborated by medical consultants were included in the study.

### Data Collection

A pre-designed data retrieval template was utilised to extract demographic and clinical attributes ranging from age, gender, marital status, occupation, year of hospital admission, the portal of in-hospital admission, duration of hospital confinement, number of concurrent illnesses (es) & specialities to outcome of hospitalisation. The substantive diagnosis made by the attending specialist was upheld and utilised in the analysis. This was further categorised, considering the affected organ systems as the World Health Organisation (WHO) recommended in its International Classification of Disease (ICD-10) protocol.^13^ Patients whose morbidity fell under cardiology, nephrology, neurology, endocrinology, dermatology, gastroenterology, haematology, non-infectious general medicine and pulmonology were stratified under the non-infectious disease group. In contrast, patients with human immunodeficiency virus (HIV), tuberculosis, viral haemorrhagic fever, hepatitis and viral pneumonia were stratified under the infectious disease group.

### Data Analysis

Analysis of the data obtained was undertaken using Statistical Product and Service Solutions (SPSS) version 25 (IBM Inc.). Qualitative variables were summarised using frequencies and percentages, while mean and standard deviation [expressed as mean (SD)] were employed for quantitative variables. A comparison between categorical variables was undertaken using Chi-square, while the Student T-test was utilised to measure continuous variables. Gender stratification of specific clinico-demographic attributes was undertaken, and appropriate comparisons were made. Regression analyses were carried out to determine the predictive ability of specific clinico-demographic parameters on the duration of hospital confinement and outcome with the adjusted odds ratio (AOR) interpreted and p-value set at < 0.05. Kaplan Meiers survival plots of effects of distinct demographic and clinical parameters on outcome were depicted. Modifiers of mortality at 30 days on Cox regression were highlighted. The approval for the study was granted by the Ethics and Research Committee of UniOsun Teaching Osogbo, Nigeria, with the reference number - UTH/REC/2024/06/951

## Results

**Figure 1:**
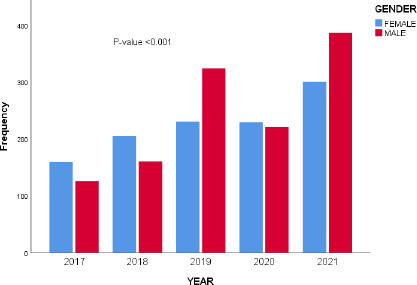
Yearly distribution of admission by gender

A total of 2340 patients were confined within the medical wards throughout the 5 years under scrutiny, with the male and female respondents accounting for 52% (n=1217) and 48% (n=1123), respectively. The male-to-female ratio was 1.1:1. The majority of the hospital confinements occurred in 2021 (n=686,29.3%), while years 2017, 2018, 2019, and 2020 accounted for 12.2%, 15.6%, 23.7%, and 19.2%, respectively. The yearly variation in the gender of admitted medical patients was statistically significant, p < 0.001 (Figure 1). The hospitalised patients' ages ranged between 16 and 108 years, with a mean age of 53.2(18.3)years. The predominant age group affected was 45-64 years (n=875, 37.3%), with male preponderance across all the age groups.

Male patients were more likely to be married (X^2^ = 81.16, p-value <0.001), had more exposure to non-infectious aetiologies (X^2^= 47.07, p-value < 0.001), and were more likely to be admitted via the accident and emergency unit (X^2^=58.291, p-value <0.001), likely to have more co-morbid conditions (X^2^= 49.86, p-value <0.001) and to be discharged home (X^2^= 11.89, p-value 0.018), The female respondents were more likely to be traders (X^2^ =431.7, p-value-<0.001).

The proportion of admitted elderly patients was significantly higher as the years progressed than the study commencement years (p-0.012). The other demographic and clinical attributes are presented in Table 1. The mean duration on admission was 8.17 (7.9) days and was statistically higher in the female patients compared to the male counterparts (p-<0.001). More patients spent ≤ 7 days on admission (n=1412,60.7%).

**Table 1:** Gender categorisation of demographic and clinical parameters of medically confined patients

Variables	Male n (%)	Female n (%)	Total n (%)	X^2^	P-value
**Age (yrs)**	53.0 (18.3)	53.3 (18.4)	53.2 (18.3)	0.167	0.692
**Age group (years)**					
**<18**	31 (1.3)	27 (1.2)	58 (2.5)	1.233	0.753
**18-44**	365 (16.6)	334 (14.3)	699 (29.9)		
**45-64**	465 (19.9)	410 (17.5)	875 (37.3)		
**>65**	356 (15.3)	352 (15.1)	708 (30.0)		
**Total**	1217 (52.0)	1123 (48.0)	2340 (100.0)		
**Marital status**					
**Single**	116 (5.0)	93 (3.9)	209 (8.9)	81.158	< 0.001*
**Married**	1097 (46.9)	955 (40.8)	2052 (87.7)		
**Widow**	3 (0.1)	72 (3.1)	75 (3.2)		
**Divorced**	1 (0)	3 (0.2)	4 (0.2)		
**Total**	1217 (52.0)	1123(48.0)	2340 (100)		
**Occupation**					
**Civil servant**	243 (10.4)	173(7.3)	416 (17.7)	431.712	<0.001*
**Trader**	248 (10.6)	610 (26.0)	858 (36.6)		
**Artisan**	274 (11.6)	64 (2.7)	338 (14.3)		
**Student**	107 (4.5)	88 (3.8)	195 (8.3)		
**Self employed**	39 (1.7)	14 (0.6)	53 (2.3)		
**Retiree**	217 (9.3)	92 (3.9)	309 (13.2)		
**Dependant**	79 (3.3)	77 (3.3)	156 (6.6)		
**Unemployed**	10 (0.4)	5 (0.2)	2340 (100)		
**Infectious/Non-infectious**					
**Non-infectious**	987(42.2)	953 (40.7)	1940 (82.9)	47.077	< 0.001*
**Infectious**	109 (4.7)	135 (5.8)	244 (10.4)		
**Not stated**	121 (5.2)	35 (1.5)	156 (6.7)		
**Portal of admission**					
**A&E**	1133 (48.4)	1037 (44.3)	2170 (92.7)	59.291	<0.001*
**MOPD**	26 (1.1)	52 (2.2)	78 (3.3)		
**HOPD**	2 (0.1)	3 (0.1)	5 (0.2)		
**Renal unit**	52 (2.1)	26 (1.1)	78 (3.3)		
**Holding Bay**	6 (0.3)	5 (0.2)	11 (0.5)		
**Nos of Specialties**					
**None**	121 (5.2)	35 (1.5)	156 (6.7)	49.875	<0.001*
**1**	980 (42.0)	928 (39.7)	1908 (81.5)		
**2**	110 (4.7)	155 (6.6)	265 (11.3)		
**3**	6 (0.3)	5 (0.2)	11 (0.5)		
**Duration of confinement(days)**	7.54±6.6	8.86 ± 8.1	8.17± 7.9	16.543	<0.001*
**Outcome**					
**Discharged**	901 (38.5)	803 (34.3)	1703 (72.8)	11.896	0.018*
**DAMA**	97(4.1)	103 (4.4)	200(8.5)		
**Referred**	39(1.7)	52 (2.2)	94 (3.9)		
**Transferred out**	1	9 (0.4)	10 (0.4)		
**Dead**	179(7.6)	156 (6.7)	335(14.3)		

* Statistically significant at p-value < 0.05, A&E- Accident & Emergency, MOPD-Medical Outpatient Department, HOPD-Haematology Outpatient Department, DAMA- Discharged against medical advice.

Non-infectious diseases predominated as the principal cause of hospitalisation in 82.9% with a significantly prolonged period of confinement compared to patients with infectious diseases (8.2(8.01) days vs 7.9 (7.2) days). The preponderant non-infectious morbidities were cerebrovascular accidents (n=316, 13.5%), acute kidney injury on a background chronic kidney disease (n=271, 11.6%), and type 2 diabetes mellitus (n=140, 6.0%). In the same vein, the predominant infectious morbidities were pulmonary tuberculosis (n=102,41.0%), HIV infection (n=31,12.7%) and chronic hepatitis B viral infection (n=23,9.4%).

Cerebrovascular accidents (CVA) affected the elderly (> 65 years) more (n=158,50.2%), unlike pulmonary TB (n=51, 50%), which was more widespread among the young (18-44 years). Infectious morbidities were significantly more pronounced among the female while the male gender had a higher burden of non-infectious morbidities (p<0.001). Non-infection morbidities significantly affected the age group 45-64 years (42.2% vs 31.2%, p< 0.001) more when compared to infectious morbidities.

The medical sub-specialities with the highest patient population were neurology (n=481, 20.6%), nephrology (n=406, 17.4%), endocrinology (n=331, 14.1%) and cardiology (n=305, 13.0%) while the specialties with the least patients' load were haematology (n=61,2.6%) and dermatology (n=11,0.5%) (Table 2). The documentation of medical diagnosis in 6.5% of the admitted respondents (n=152) was missing. The gender stratification of the clinical diagnosis across sub-specialties was statistically significant (p <0.001). Morbidities under pulmonology (p-0.576), haematology (p-0.355) and general medicine (p-0.770) were not significantly affected by gender (Table 2). Female patients had a more pronounced burden of cardiology, endocrinology and pulmonology morbidities.

**Table 2:** Gender stratification of morbidities diagnoses across medical sub-specialties from January 2017 to December 2021. Nigeria (n=2340)

Specialty	Total n (%)	Male n (%)	Female n (%)	Mean age (SD) years	Mean Duration (SD) days	X^2^	P-value
**Neurology**	481 (20.6)	260 (11.1)	221 (9.4)	57.6 (18.3)	7.7 (6.9)	19.482	0.003*
**Stroke (CVA)**	316 (65.7)	161 (33.5)	155 (32.2)				
**Meningitis**	32 (6.7)	16 (3.3)	16 (3.3)				
**Seizure**	27 (5.6)	13 (2.7)	14 (2.9)				
**Tetanus**	28 (5.8)	26 (5.4)	2 (0.4)				
**Delirium**	25 (5.2)	14 (2.9)	11 (2.3)				
**Movement disorders**	19 (4.0)	12 (2.5)	7 (1.5)				
**Others**	34 (7.1)	18 (3.7)	16 (3.3)				
**Nephrology**	406 (17.4)	238 (10.2)	168 (7.2)	50.1 (18.9)	8.5 (7.8)	22.923	<0.001*
**AKI**	50 (12.3)	34 (8.4)	16 (3.9)				
**A/CKD**	271 (66.7)	172 (42.4)	99 (24.4)				
**GN**	14 (3.4)	7 (1.7)	7 (1.7)				
**Pyelonephritis**	25 (6.2)	6 (1.5)	19 (4.7)				
**UTI**	31 (7.6)	13 (3.2)	18 (4.4)				
**Others**	15 (3.7)	6 (1.5)	9 (2.2)				
**Endocrinology**	331 (14.1)	128 (5.5)	203 (8.7)	56.4 (15.3)	9.9 (9.5)	14.549	0.037*
**Type 2 DM**	140 (42.3)	53 (16.0)	87 (26.3)				
**HHS**	65 (19.6)	19 (5.7)	46 (13.9)				
**DKA**	12 (3.6)	8 (2.4)	4 (1.2)				
**DMFS**	67 (20.2)	32 (9.7)	35 (10.5)				
**Hypoglyceamia**	17(5.1)	7 (2.1)	10 (3.0)				
**Thyrotoxicosis**	9 (2.7)	1 (0.3)	8 (2.4)				
**Type 1 DM**	4 (1.2)	3 (0.9)	1 (0.3)				
**Others**	17 (5.1)	5 (1.5)	12 (3.6)				
**Cardiology**	305 (13.0)	151 (6.5)	154 (6.6)	57.2 (16.1)	7.7 (8.7)	19.075	<0.001*
**HTN/HHDX**	84 (27.5)	55 (18.0)	29 (9.5)				
**Arrythmia**	2 (0.7)	0	2 (0.7)				
**CCF 2^0^ HHDX**	167 (54.8)	75 (24.6)	92 (30.2)				
**CCF 2^0^ DCM**	32 (10.5)	17 (5.6)	15 (4.9)				
**ACS**	5 (1.6)	1 (0.3)	4 (1.3)				
**Others**	15 (4.9)	3 (1.0)	12 (3.9)				
**Respiratory**	252 (10.8)	110 (4.7)	142 (6.1)	53.3 (18.6)	8.6 (8.4)	3.923	0.576
**PTB**	102 (40.5)	42 (16.7)	60 (23.8)				
**Pneumonia**	83 (32.9)	38 (15.1)	45 (17.9)				
**COPD**	32 (12.7)	18 (7.1)	14 (5.6)				
**Asthma**	12 (4.8)	4 (1.6)	8 (3.2)				
**Lung Cancer**	10 (4.0)	4 (1.6)	6 (2.4)				
**Others**	13 (5.2)	4 (1.6)	9 (3.6)				
**Gastroenterology**	235 (10.0)	133 (5.6)	102 (4.4)	46.3(17.3)	6.8 (5.1)	39.300	<0.001*
**PUD**	27 (11.5)	6 (2.6)	21 (8.9)				
**CLD 2^0^ CHB**	78 (33.2)	53 (22.6)	25 (10.6)				
**PLCC/HCV**	26 (11.1)	14 (6.0)	12 (5.1)				
**Liver Cirrhosis**	17 (7.2)	14 (6.0)	3 (1.2)				
**Gastroenteritis**	44 (18.7)	16 (6.8)	28 (11.9)				
**Upper GI bleed**	22 (9.4)	17 (7.2)	5 (2.1)				
**Others**	21 (8.9)	13 (5.5)	8 (3.4)				
**General Medicine**	102 (4.4)	40 (1.8)	62 (2.6)	49.3(20.3)	6.7 (8.0)	2.255^#^	0.770
**Snake bite**	3 (2.9)	1 (1.0)	2 (1.9)				
**Malaria**	19 (18.6)	8 (7.8)	11 (10.8)				
**Organophosphate-poisoning**	2 (2.0)	1 (1.0)	1 (1.0)				
**HIV/AIDS**	32 (31.4)	10 (9.8)	22 (21.6)				
**Sepsis Syndrome**	45 (44.1)	19 (18.6)	26 (25.5)				
**Haematology**	61 (2.6)	32 (1.4)	29 (1.2)	37.2 (20.9)	7.8 (7.7)	2.895	0.355
**Sickle cell crisis**	31 (50.8)	14 (23.0)	17 (27.8)				
**Deep venous thromboses**	2 (3.3)	2 (3.3)	0				
**Lymphoproliferative Disease/Cancer**	24 (39.3)	13 (21.3)	11 (18.0)				
**G6PD Deficiency**	4 (6.6)	3 (4.9)	1 (1.6)				
**Dermatology**	11 (0.5)	4 (0.2)	7 (0.3)	47.1 (22.9)	7.2 (7.5)	9.791^#^	0.010*
**Herpes Zooster**	3 (27.3)	3 (27.3)	0				
**HIV Dermopathy**	1 (9.1)	0	1 (9.1)				
**Scleroderma**	1 (9.1)	1 (9.1)	0				
**Bullous Impetigo**	1 (9.1)	0	1 (9.1)				
**Erythroderma**	5 (45.5)	0	5 (45.5)				
**Diagnosis not stated**	156 (6.7)	121 (5.2)	35 (1.5)				

* Statistically significant at p < 0.05

# Fischer Exact value, CVA Cerebrovascular accident, AKI- Acute Kidney Injury, A/CKD- Acute on chronic kidney disease, GN- Glomerulonephritis, UTI- Urinary tract infection, DM- Diabetes mellitus, HHS-Hyperglycaemic-hyperosmolar state, DKA-Diabetic keto-acidosis, DMFS-Diabetes mellitus foot syndrome, HTN/HHDX- Hypertension/hypertensive heart disease, CCF- Congestive cardiac failure, DCM-Dilated cardiomyopathy, ACS- Acute coronary syndrome, PTB- Pulmonary tuberculosis, COPD- Chronic obstructive airway disease, PUD-Peptic ulcer disease, CLD- Chronic liver disease, CHB- Chronic hepatitis B, PLCC-Primary liver cell cancer, HCV- Hepatitis C virus, GI-Gastrointestinal, HIV/AIDS- Human Immunodeficiency virus/Acquired immunodeficiency syndrome

Table 2 depicts the gender variations in the mean ages and duration of confinement across medical subspecialisations. Neurology and haematology subspecialties had the highest (57.6(18.3) years) and lowest mean ages (37.2 (20.9) years), respectively. For the duration of the study, 335 (14.3%) mortalities were recorded, with the highest number (n=96, 28.7%) observed in the year 2019 and the lowest (n=43, 12.8%) in the year 2017. Mortalities were preponderant among male patients (n=179,53.4%) compared to female patients (n=156, 46.6%).

Male and female hospitalised respondents had crude mortality rates of 7.64% and 6.67%, respectively. The yearly distribution of mortalities across genders was statistically significant (p=0.029).

Non-infection-attributable mortalities (n=294,87.7%) were statistically more apparent than infectious-attributable mortalities (n=37,11.0%) (p<0.001). The commonest non-infectious causes of mortalities were CVA (n=68,23.1%), A/CKD (n=44,15%) and type 2 DM (n=25, 8.5%), while PTB (n=17,7.0%) and HIV/AIDS (n=5,2.1%) predominated for infectious diseases. (Table 3) Mortality was most pronounced among the middleaged group. The type of medical diagnosis (p<0.001) and medical sub-specialty involved (p<0.001) had a significant influence on mortality.

**Table 3:** Gender stratification of mortalities across years & medical sub-specialties from January 2017 to December 2021. Nigeria (n=2340)

Variable	Total admission n (%)	Total mortalities n (%)	Male n (%)	Female n (%)	Mean Age (SD) years	X^2^	P-value
**Year**							
**2017**	285 (12.2)	43 (12.8)	22 (6.6)	21 (6.2)	54.3 (19.7)	10.706	0.029*
**2018**	365 (15.6)	51 (15.2)	30 (9.0)	21 (6.2)	56.0 (16.5)		
**2019**	554 (23.7)	96 (28.7)	62 (18.5)	34 (10.1)	54.0 (19.4)		
**2020**	450 (19.2)	92 (27.5)	44 (13.1)	48 (14.3)	57.7 (19.3)		
**2021**	686 (29.3)	53 (15.8)	21 (6.3)	32 (9.6)	56.9 (16.5)		
**Total**	2340 (100)	335 (100)	179 (53.4)	156 (46.6)	55.8 (18.5)		
**Age-groups(years)**							
**<18**	58 (5.2)	7 (0.3)	4 (1.2)	3 (0.9)		0.971	0.258
**18-44**	699 (29.9)	84 (3.6)	43 (12.8)	41 (12.2)			
**45-64**	875 (37.3)	127 (5.4)	69 (20.6)	58 (17.3)			
**≥ 65**	708 (30.3)	117 (5.0)	63 (18.8)	54 (16.1)			
**Total**	2340 (100)	335 (14.3)	179 (53.4)	156 (46.6)			
**Specialty**							
**Cardiology**	305 (13.0)	30 (9.0)	12 (3.6)	18 (5.4)	62.7 (16.5)	94.342	<0.001*
**Neurology**	481 (20.6)	91 (27.2)	47 (14.0)	44 (13.1)	60.2 (17.2)		
**Nephrology**	406 (17.4)	66 (19.7)	38 (11.3)	28 (8.4)	52.8 (20.1)		
**Endocrinology**	331 (14.1)	39 (11.6)	17 (5.1)	22 (6.6)	57.2 (14.8)		
**Gastroentero**	235 (10.0)	42 (12.5)	34 (10.1)	8 (2.4)	48.5 (18.4)		
**Dermatology**	11 (0.5)	1 (0.3)	0	1 (0.3)	42.0		
**Pulmonology**	252 (10.8)	37 (11.7)	18 (5.4)	19 (5.7)	56.5 (17.9		
**General medicine**	102 (4.4)	10 (3.0)	5 (1.5)	5 (1.5)	58.2 (23.8)		
**Haematology**	61 (2.6)	10 (3.0)	4 (1.2)	6 (1.8)	45.0 (22.8)		
**Not Stated**	156 (6.7)	4 (1.2)	2 (0.6)	2 (0.6)	60.3 (12.7)		

* Statistically significant at p < 0.05

**Figure 2 (a-f):**
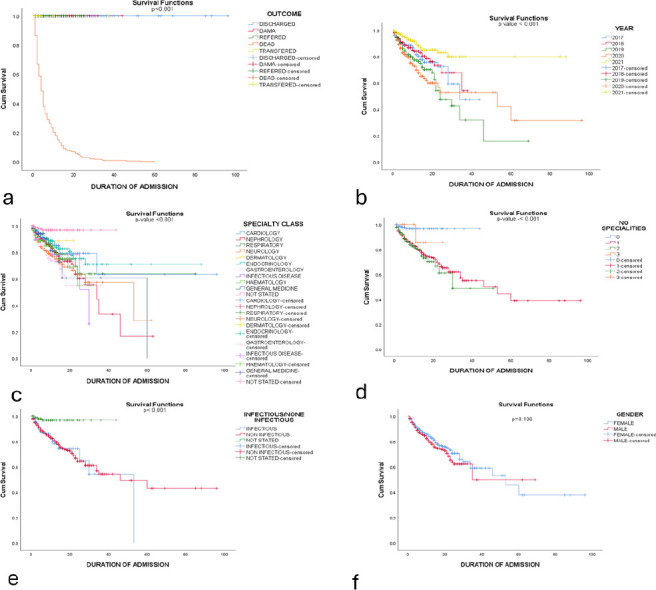
Kaplan Meier's plots in hospitalised patients reflecting effects of specific clinical attributes on survival

Multiple regression analysis carried out to predict the association between age, age group, diagnosis, type of medical speciality, number of concurrent medical illnesses & classification into infectious/non-infectious aetiologies on the duration of hospital confinement was not statistically significant F (6,2166) =0.089, p=0.563, R^2^=0.002. Using multivariate regression analysis, increasing age, duration of confinement, and disease classification into infectious/non-infectious significantly influenced the clinical outcomes. Increasing age was associated with a decreased likelihood of unfavourable clinical outcome (AHR- 0.9 95% CI 0.9-1.2; P-0.004) while increasing duration of confinement (AHR-1.03, 95% CI 1.01-1.05; P=0.002) and infectious disease diagnosis (AHR 1.4, 95%CI 1.02-1.95; P=0.04) were associated with higher risk of unfavourable clinical outcome. Gender (p-0.697) and the number of medical specialties involved in morbidity (p-0.087) did not statistically influence clinical outcomes. Survival of the medically confined patients was significantly affected by the number of specialties involved (p<0.001), specialty affected (p<0.001), clinical outcome (p <0.001), stratification into infectious/non-infectious disease (p<0.001) and yearly trend of hospitalisation (p-<0.001) on Kaplan Meier graphs (Figure 2)

Cox survival analysis significantly demonstrated protective effects of gender (AHR 0.5, 95% CI 0.45-1.61; P<0.001) and increasing duration on confinement (AHR 0.94,95% CI 0.92-0.95; P<0.001) on mortality at 30 days. However, mortality risk at 30 days increased by 1.4-fold as the number of medical specialties involved increased (p=0.006). Poor clinical outcome (p<0.001) culminated in a 6.2-fold increase in the risk of mortality at 30 days (Table 4).

**Table 4:** Cox survival regression on the effects of specific clinical attributes on mortality odds

Variable	β	P-value	Exp β	95% CI for Lower	Exp β Upper
**Specialty**	-0.21	0.390	0.980	0.935	1.027
**Infectious/Non-infectious**	0.176	0.244	1.193	0.886	1.605
**Number of Specialties**	0.388	0.006*	1.474	1.115	1.948
**Gender**	0.561	<0.001*	0.571	0.455	0.717
**Duration on confinement**	0.061	<0.001*	0.940	0.922	0.959
**Clinical outcome**	1.837	<0.001*	6.277	5.558	7.152

* Statistically significant at p < 0.05

## Discussion

The retrospective evaluation of the cardiovascular risk(s) pattern, outcome modifiers and survival peculiarities among medically confined patients over a 5-year time frame, as highlighted, was embarked upon to have a tropical perspective of the relentlessly evolving global trend of medical hospitalisation. The swing in the gender pendulum towards the male sex, which was non-static throughout the study duration, corroborated over a decade-old similar finding by Okunola et al.^9^ in our facility and other parts of Nigeria.^10^,^12^ Male gender predominance among medical in-patients has been substantiated in other parts of the African continent with limited findings of female predominance, which may be related to the heavy burden of care bore by the male gender with increased odds of morbidities, better economic capacities influencing prompt hospital attendance and inculcation of unwholesome lifestyle habits and tendencies which is the fulcrum of the ever-increasing burden of non-remitting medical morbidities.^6^,^7^,^8^,^14^,^15^

The preponderant morbidities among medical in-patients highlighted in this survey were majorly non-infectious, namely cerebrovascular accidents (CVD), CKD, CCF and type 2 DM, which agrees with prior findings in our facility. However, DM and CKD were previously 2^nd^ and 3^rd^ respectively.^9^ Tuberculosis still maintained its dominance as the most frequent infectious morbidity in the studied population, occupying the 6^th^ position as opposed to the second position it previously occupied. The dwindling burden of tuberculosis in this study, as opposed to the previous finding in this facility, despite the increase in the study duration, might not be unconnected to the persistent local and global rise in the burden of non-infectious chronic medical morbidities.^6^,^9^,^10^,^12^,^14^

Infectious diseases, however, are still on the rise in certain parts of Africa where HIV infection and severe pneumonia predominated medical confinement. 7,8,15 During the 5-year survey, the stepwise continuous surge in the prevalence of non-infectious chronic medical morbidities and the declining proportion of infectious disease further gives credence to sub-optimal/non-existent preventive approaches to effectively curtail cardiovascular hazards in our clime.^12^

The high frequency of hospitalisation bore by age groups 45-64 years (37.3%) and ≥65 years (30.3%) were at variance with previous observations where the elderly accounted for 29.8% and 27.3% of the total male and female confinements, respectively but compares favourably with 43.5% documented in a Southern state of the country.^9^, affectation of the age group 15-44 years was reported to be the most prevalent among the medical inpatients in Calabar, a coastal city in Nigeria, with a frequency of 37.9%.^16^

The increased quota of hospitalisation in older adults has also been documented in Ado-Ekiti, another southern state in Nigeria and other parts of Africa.^12^,^14^ The mean age of the population (53.2 (18.3) years) is higher than 38 years and 43 years reported in Uganda and Northern Ethiopia, respectively, but close to 50.9 (19.8) years reported by Adekunle et al.^12^ also in Nigeria.^8^,^14^ The divergent age-group affectation may reflect the population dynamics of the studied locations.

The predominant affectation of the middle age group, which represents the working cohort, as highlighted in this study, is quite worrisome. It has the potential to reduce gross domestic earnings, hence the need for timely interventional strategies to address the menace.

A significant percentage of the patients studied (60.7%) were confined for ≤ 7 days, similar to the observation in Northern Ethiopia.^14^ The duration of hospital confinement was significantly longer for females than for males in this study, which is at variance with previous findings in the same region of Nigeria.^12^ The overall mean duration of confinement was 8.2(7.8) days, with patients having non-infectious morbidities experiencing protracted confinement compared with their counterparts with infectious morbidities, which may be explained by the underlying relentless pathophysiological bases of the divergent but interrelated cardiovascular hazard(s) and their sequelae.

The duration of confinement in this survey was most protracted in patients with endocrine disorders (9.9(9.5) days), respiratory disease (8.6(8.4) days), and nephrology disorders (8.5(7.8) days). This contrasts to lengthened hospitalisation from neurological and infectious diseases, as Adekunle et al.^12^ reported with previous validation.^17^ Non-infectious morbidities were significantly predominant (87.2%) among those confined for > 4 weeks, principally with A/CKD. This is higher than 66.7%, as reported by Hailu et al.^14^, among 2084 hospitalised patients in Northern Ethiopia, although cardiovascular diseases (CVD), encompassing CKD, were the culprit.

Cumulatively, the prevalent morbidities among the deceased were CVA and A/CKD for non-infectious illnesses and PTB and CHB virus-induced chronic liver disease (CLD) for infectious illnesses. Cerebrovascular accident (21.5%), type 2 DM (17.8%) and A/CKD (16.2%) were morbidities with the highest case fatality values among non-infectious conditions. In comparison, PTB (16.7%) and CHB-induced CLD had the highest case fatality proportions among infectious illnesses. A high case fatality figure for CVD was also reported in Cameroon.^18^ The exaggerated fatalities may be due to poor knowledge of disease symptoms, late hospital presentation, huge disease burden and limited financial capability. The causes of death were not documented in an earlier study in this centre. The staggering proportion of mortalities attributed to non-infectious disease, as demonstrated in this study, has been reported in some parts of Nigeria and Africa in varying amounts.^7^, ^8^,^10^, ^14^, ^19^ Although CVA was the predominant non-infectious aetiology for mortality, heart failure (15.1%), and malignancy (21.7%) were the causes of death in Ethiopia and Sudan, respectively.^7^,^14^

Infectious mortality predominated 44.5% of the cases in a 4-year retrospective evaluation carried out in Uganda.^8^ Over a 50-year period from the mid-fifties, infectious mortality predominantly accounted for 17.1% of all deaths out of the 9695 patients reviewed from 15 surveys.^15^

Cerebrovascular accident, a component of CVD which has been adjudged as the penultimate global cause of mortality, is responsible for up to one-tenth of the deaths in emerging countries, particularly in Africa, with a rapidly increasing proportion due to environmental, genetic and habitual culinary practices.^4^,^5^,^20^ Individualised and collective approach by governments domiciled in highly susceptible climes remains paramount to halt the rising burden of infectious and non-infectious morbidities. The overall crude mortality rate of 14.3% is lower than the 20.8% reported in an earlier study but higher than the 4.5% reported by Okoroiwu et al.^16^ in another Southern state in the country, and this may be due to the larger sample size (49,287 vs 2340) and span (6yrs vs 5yrs) of the survey.^9^ In-hospital mortality with gender lopsidedness has been widely reported and may result from morbidity-specific and individual patients & health facility dynamics.^12^,^16^,^20^ There were significant initial surges in yearly mortality percentages with the latter decline. Relatable findings have been reported around the African continent.^12^,^15^,^16^ Fatality was more pronounced among the age group 45-64 years, which contrasted with the age group 16-44 years as reported by Okoroiwu et al.^16^ in Calabar and may largely be due to divergent medical morbidities across the age-groups with the inclusion of children.

Age, duration of hospital confinement and disease stratification into infectious/non-infectious types significantly predicted clinical outcomes in this study. A similar finding was documented in Uganda, although the duration of confinement did not predict clinical outcomes in that study. Mortality was significantly influenced by the density of co-morbid conditions, the affected specialty, the year and confinement duration, and the survival analysis's clinical outcome. These findings were difficult to corroborate as the significant paucity of data/literature exists in this area. However, it suffices to state that the lack of effective government insurance schemes, with almost, if not all, the patients paying for their medical care alone, could be a reason for mortality in this study. The survey was limited because of its retrospective form with missing patients' information, a hospital-based study, which may not truly represent the community and nonpostmortem-based final diagnosis, which may have resulted in under/over-reporting of specific causes of mortality.

## Conclusion

The predominance of non-infectious chronic debilitating medical illnesses (CVA, A/CKD & type 2 DM) as precursors of medical confinements and mortalities with predilection for age group 45-64 years over the 5-year span as reflected in this study should be a source of concern for health care practitioners and administrators. Concerted attempts targeted at effective preventive strategies across all geographical locations and strata of health facilities should be adopted by all tiers of government, particularly in Sub-Saharan Africa, to arrest this ugly pattern. Healthcare delivery should be made accessible and within reach of the populace to prevent self-medication and the use of un-tested herbal remedies, which further potentiates tendencies for protracted morbidities and mortality and delayed arrival at approved healthcare facilities. Continuous strategies to curtail infectious disease propagation should also be sustained.
